# Permeable Properties of Hygienic Nonwovens Bonded Using Mechanical, Chemical, and Thermal Techniques

**DOI:** 10.3390/polym16081132

**Published:** 2024-04-17

**Authors:** Dunja Šajn Gorjanc, Klara Kostajnšek

**Affiliations:** Department of Textiles, Graphic Arts and Design, Faculty of Natural Sciences and Engineering, University of Ljubljana, Snežniška 5, 1000 Ljubljana, Slovenia; klara.kostajnsek@ntf.uni-lj.si

**Keywords:** nonwoven fabrics, web bonding, mechanical properties, air permeability, porosity

## Abstract

The demand for hygienic nonwovens has increased, especially since 2020. As expected, the market for nonwovens showed an increase during the COVID-19 outbreak, especially in the medical and hygienic nonwovens sector. The aim of this study is to analyse the influence of the permeability properties of hygienic nonwovens that have been mechanically, chemically, and thermally bonded. Hygienic nonwovens are lightweight (from 20 to 120 g/m^2^), produced by carding (roller carding), and are bonded using three different bonding processes (mechanical, thermal, and chemical). Hygienic nonwovens are intended for protective clothing in hospitals. For the experimental part, the seven different nonwovens used for hygienic purposes were produced using the dry laying process. The samples were produced in Tosama, a factory for sanitary supplies. The research results show that the nonwoven bonding processes have a significant influence on the structural, mechanical, and permeability properties.

## 1. Introduction

Nonwovens are also known as technical fabrics made from fibres. Nonwovens are produced using high-speed and low-cost processes. They are used in a wide range of consumer and industrial products, either in combination with other materials or alone.

Nonwovens can be divided into nine types depending on the manufacturing process [1,2] as follows: drylaid—carded, drylaid—high loft (short fibre), airlaid, wetlaid, spunlaid, and meltblown, as well as being thermally, mechanically, and chemically bonded. The nonwovens analysed in this study are drylaid—carded and are thermally, mechanically, and chemically bonded.

As part of our research, we have investigated the permeable properties of hygienic nonwovens that have been mechanically, chemically, and thermally bonded. The hygienic nonwovens are lightweight (from 20 to 120 g/m^2^), produced by carding (roller carding), and are bonded using three different bonding methods (mechanical, thermal, and chemical). Hygienic nonwovens are intended for protective clothing in hospitals. In a continuation of the current research, the influence of the bonding technique on the permeability properties of the hygiene web will be investigated.

Numerous studies have been carried out on the permeability properties of hygienic nonwovens. Lui, M. et al. [3] investigated the comfort and antimicrobial properties of polyimide (PI) composite spunlace hygiene nonwovens. Among other things, the study found that increasing the plasma treatment performance had no significant effect on the permeability properties of the nonwovens. The permeability properties of fabrics are mainly related to the pore size and porosity of the fibres, which do not change before and after plasma treatment [3].

And also, the increased roughness of the fibre’s surface after plasma treatment or other treatments (chemical and thermal bonding) increases the breaking stress [3].

Venkataraman, D. et al. [4], on the other hand, deal with the further development of nonwovens in personal protective equipment. The permeability properties of PPE are closely related to the lower surface area and the lower number of fibres in the structure, which ensure higher air permeability and thermal permeability [4].

The authors present the development of hygienic nonwovens mainly used for protective equipment after the COVID-19 pandemic. Korkmaz, G. et al. [5] compared the performance characteristics of spunbonded nonwovens used as face masks. They focused on the air permeability of spunbonded and spunbonded–meltblown–spunbonded masks (SMS). They found that the air permeability of the spunbonded samples was higher. The reason for this is the smaller surface area compared to the SMS nonwoven, which consists of three layers, which results in a larger surface area and lower air permeability [5].

Somogy Skoc, M. et al. [6] are working on the development and characterization of sustainable coatings on cellulose fabrics and nonwovens for medical applications. Some of the latest research is on the use of natural waste fibres blended with polylactic acid (PLA): Kapok and cattail fibres, which are used for medical textile nonwovens [7]. The research results show that the admixture of natural fibres can reduce the mechanical properties of nonwovens, but increase the permeability properties.

### 1.1. Hygiene Nonwovens

Medical textiles or hygienic textiles are all types of textiles used in personal care, medical, and medical-related fields. They account for the largest share of production. They are used to produce diapers, hygiene products (pads and tampons), incontinence textiles, various disposable underwear, sanitary napkins, etc. [8].

The most important property of hygiene nonwovens is the absorbency of the materials and their permeability. Hygienic materials must be very soft and retain their shape even after use. For disposable diapers, for example, it is very desirable that they have soft, firm nonwoven components such as tops or backs (also called outer covers). They must also have very good protective properties. The most important requirements for hygiene textiles are absorbency, durability, flexibility, softness, and sometimes also biostability or biodegradability.

The fibres used in the medical field range from cotton, silk, and regenerated cellulose (absorbent layer) to polyester, polyamide, and polyethylene fibres for surgical gowns, bedding, sheets, pillowcases, uniforms, surgical stockings, drapes, surgical drapes, protective clothing, glass fibres used mainly for caps, masks, etc. [8].

Cotton fibres are commonly used for surgical clothing, bedding, and medical uniforms; viscose fibres are used for medical caps, masks, diapers, and handkerchiefs. Man-made fibres such as polyester fibres are used for masks, surgical headgear, and blankets; polyamide fibres are used for surgical socks; polypropylene fibres are used for protective clothing, liners, and diapers; and polyethylene fibres are used for surgical drapes [8,9,10,11,12,13,14,15,16,17].

### 1.2. Technology Process of Web Bonding of Hygiene Nonwovens

For a good adhesion between the fibres in the nonwoven, the following methods for bonding the base layer have been established:

- mechanical process (needling, felting, water jet, and quilting),
- chemical process (impregnation, printing, or spraying with binder-glue),
- thermal process (melting of the binder fibre coating),
- combined processes.

For most mechanically bonded nonwovens, needling and hydroentanglement are encountered in hygiene textiles.

Needling—nonwovens are fatigued by piercing many needles. Depending on the intensity, a distinction is made between pre-needling and needling.

The points at which the needles pierce the pre-needled, layered canvas are anchor points around which most of the fibres reorient during further bonding of the canvas textile during stretching from the transverse to the longitudinal direction. Bonding with a water jet—nonwovens bonded with a water jet (spunlace) are also called hydroentangled or hydraulically needled textiles. The bonding of sanitary nonwovens is achieved using the action of several jets of water on the horizontal nonwoven fabric. The water is under a pressure of 10 to 60 MPa just before it exits the jets [17].

The chemical bonding of nonwovens is carried out with the help of the formation of adhesive sites. These sites are formed by the bonding of the fibre surface with the binder (adhesive). When bonding with binders, it is very important to know the composition and the form of the bond, because the chemical composition determines the adhesion strength, mechanical properties, heat resistance, and hygienic properties of the textiles.

To produce nonwovens, binders are used in the following forms: polymer solutions, polymer pastes, polymer suspensions/dispersions, and foamy polymer suspensions/dispersions.

The process of bonding nonwovens with various binders (adhesives) includes the following steps: establishing contact between the fibre surface and the binder, wetting the fibre surface with a binder, bonding (activation) of the binder-binding part of the fibres together, migration of the binder into the cotton textile, bonding of the binder, and formation of a stable and durable bond between the fibres [1,17].

Thermally bonded nonwovens are textiles made by melting thermoplastic binders or the fibres themselves (polyester, polypropylene, etc.) using heat.

Several types of thermal bonding are established, as follows:

Calendering—in this process, the nonwoven fabric is passed through two heating rollers that are under a certain pressure. During this process, the fibres melt and are further compressed by the pressure of the rollers.

Ultrasonic bonding—in ultrasonic bonding, the process is like calendering, the difference being that instead of the top roller or drum, a device is used that generates heat using ultrasonic energy [17,18,19,20,21].

## 2. Materials and Methods

### 2.1. Materials

In the experimental part, seven nonwovens used for hygienic purposes were produced using the drylaid nonwoven process. The samples were produced in Tosama, a sanitary supplies factory, and were bonded using mechanical, thermal, and chemical bonding techniques.

This study focused on the influence of bonding techniques on the permeability properties of nonwovens, which are mainly used in hospitals for hygiene purposes. All samples were produced using the drylaid bonding process (carded nonwovens).

The investigation focused on samples that were categorized in the group of lighter nonwovens for hospital textiles. In this study, we limited ourselves to a selection of samples made from 100% cotton fibres (samples 5 and 6), from man-made fibres (100% PP—samples 2, 3, and 7), 100% viscose fibres (sample 4), and from blends of natural and man-made fibres (70% PP, 15% CV, 15% CO—sample 1).

All samples were produced using the same technological process—the carding process. We have taken this into account in the statistical analysis and discussion of the results. In this study, we focused on topical materials that are most commonly used in hospitals for hygiene in different areas (protective clothing during surgery, protection of equipment and operating tables, etc.). Since the samples do not have a specific name, we numbered them from 1 to 7.

**Figure 1:**
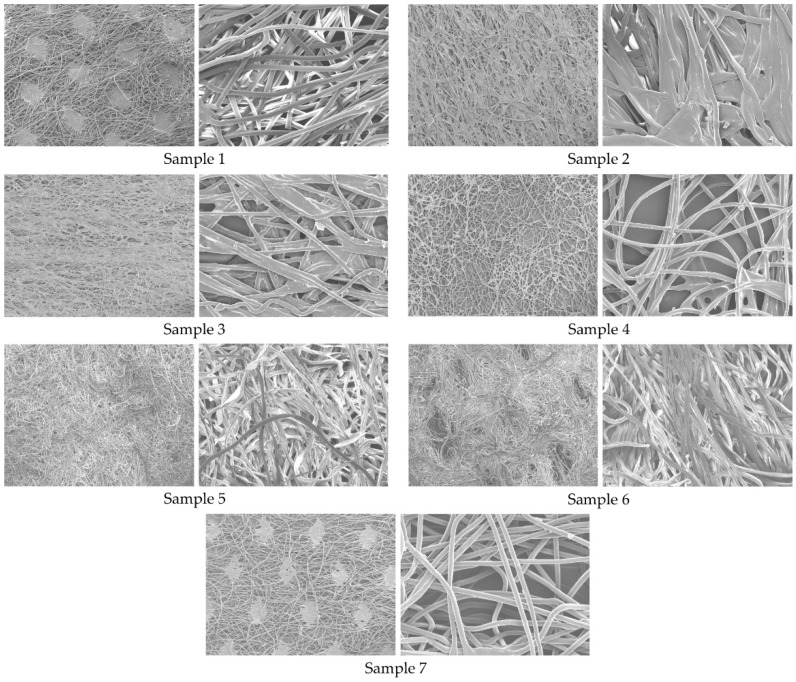
Microscopic view of the samples at 25× (**left**) and 200× (**right**) magnification.

The analysed samples are listed in Table 1. Figure 1 shows a sample of images taken with a JSM-6060 LV scanning electron microscope (SEM) (Jeol, Tokyo, Japan) at 25× magnification (Samples 1 to 7 are on the left side of the presented samples, Figure 1), where the scale bar of 1 mm on Figure 1 represents a reading of 15 μm; and at 200× magnification (Samples 1 to 7 are on the right side of the presented samples, Figure 1), where the scale bar of 5 mm on Figure 1 represents a reading of 15 μm. The average fibre diameters measured in scanning electron micrographs at 200× magnification are listed in Table 2, as well as the thickness and mass of the samples examined. The thickness and mass of the samples (Table 2) were measured according to ISO 9073 (Part 1 and Part 2) [22].

### 2.2. Methods

#### 2.2.1. Breaking Stress and Elongation

In the tensile test, the breaking stress and elongation are determined, as well as the tensile force acting on the specimen in the direction of loading at the time of fracture and the change in length of the specimen.

Breaking stress and elongation were measured on the Instron 5567 dynamometer (Instron, Bristol, UK) according to ISO 13934-1 [23]. The measured values were processed using the Blue Hill program (Instron, Bristol, UK). The program also enables the retrospective interpretation of the measured values.

#### 2.2.2. Air Permeability

The air permeability of a flat product indicates the amount of air that passes through a given surface of the test piece in a given time at a given pressure, or the air flow that passes through a given area of the fabric.

The air permeability of fabrics has a significant impact on the comfort and technical properties of clothing. Fabrics that are more air permeable tend to regulate temperature balance better and, thus, provide more comfort. The greater the porosity, the more permeable the fabric is to air [24,25,26,27].

Air permeability was measured according to the standard ISO 9237 [28] on Air-Tronic B apparatus (Mesdan, Raffa, Italy). The measurement was carried out in such a way that air at a pressure of 100 Pa flowed through a 20 cm^2^ opening, to which a sample was attached. The v l/h measured values were converted using the following Equation (1):

(1) $$Q=\frac{q}{6\cdot F}$$

where *Q*—volume of air sucked in m^3^ in 1 min converted to 1 m^2^ at 20 mm water column in m^3^/m^2^/min; *q*—air volume flowing through the filter or read value in l/h; and *F*—test surface, which is 20 cm^2^.

#### 2.2.3. Thermal Conductivity

The thermal conductivity was determined on laboratory equipment at the University of Ljubljana, Faculty of Natural Sciences and Engineering, Department of Textiles, Graphic Arts and Design, in accordance with DIN 52612 [29]. The measuring device has an insulating plate on the underside. A heating block with a temperature of 60 °C is placed on the insulating plate.

A second copper measuring plate, a glass plate with known thermal conductivity (*λ_n_* = 1.0319 Wm^−1^K^−1^), and a thinner copper measuring plate were then placed on top.

This was followed by our sample, a thicker measuring plate, and a block with a temperature of 20 °C. Altogether, we connected to a temperature measuring device with thermocouples. The temperature of the individual plates was measured every three minutes until it no longer changed [30,31,32,33]. The thermal conductivity was calculated using the following Equation (2):

(2) $$\lambda_{x}=\lambda_{n}\cdot \frac{dx}{dn}\cdot \frac{T_{3}-T_{2}}{T_{2}-T_{1}}$$

where *λ_x_* is the thermal conductivity of the test specimen (Wm^−1^K^−1^), *λ_n_* is the thermal conductivity of the reference glass pane (*λ_n_* = 1.0319 Wm^−1^K^−1^), *dx* is the thickness of the test specimen (mm), *T*_1_ is the temperature of the thin copper plate (K), *T*_2_ is the temperature of the copper plate that is in the middle (K), and *T*_3_ is the temperature of the thicker copper plate (K).

#### 2.2.4. Porosity

Hygienic nonwovens have a porous structure. This means that there are many empty air spaces between the fibres. Porosity is defined more precisely using several parameters that describe the internal geometric structure of a nonwoven [34,35].

The size and distribution of pores were determined using the Jakšič method, known as the J method. A rotameter is used to measure the volumetric velocity of air flow through a given area of the dry sample at different pressure differences [36].

The porosity-measuring device consists of a compressor that generates an overpressure of 8 bar, a pressure reducer that reduces the pressure to 2.5 bar, and an auxiliary vessel, to which a rotameter with a measuring range of up to 833 cm^3^/s and a manometer are connected. The moving air flows through the rotameter into the measuring element, to which the sample is attached. The measuring head allows the clamping of 1.5 and 10 cm long samples.

For each sample, three dry and three wet samples (soaked in distilled water) were prepared. The sample was clamped into the 1.5 cm long measuring head and the measurement was started [36].

#### 2.2.5. Surface Openness Using Image Analysis

From the images obtained during analysis of the scanning electron microscope (JSM 6060-JV, Jeol, Japan), we also obtained material for the later study of surface openness. The photographs were then analysed using the ImageJ program (NIH’s National Institute of Mental Health, Neuroscience Center Building in Rockville, MD, USA).

The ImageJ program can be used to determine the percentage of open area. For the analysis, the image must be converted to an 8-bit image and converted to binary format. Then, the program automatically selects the darker areas of the digital image that represent the surface openness (Op) of the nonwoven, which the program calculates as a percentage (%) [37,38,39,40].

#### 2.2.6. Statistical Analysis

The relationship between independent parameters such as mass, thickness, fibre diameter, surface openness, and dependent parameters (average mean pore diameter, bubble point, breaking stress, breaking elongation, air permeability, and thermal conductivity) was investigated using regression analysis. The regression analysis evaluates the strength of the relationship between independent and dependent variables.

A multiple regression model is used when more than one independent variable influences a dependent variable. When predicting the outcome variable, it is important to measure how each of the independent variables moves in its environment and how its changes affect the output or target variable.

Correlations above 0.7 are considered strong, because if R = 0.7, then R^2^ = 0.49, which means that around 50% of the variance in the response variable can be explained by the explanatory variable. The *p*-values indicate whether there is a statistically significant relationship between each predictor variable and the response variable.

A significance level of 0.05 means that it is assumed, with a risk of 5%, that there is a difference between the variables, although there may not be a difference. On the other hand, a *p*-value of more than 0.05 means weak evidence, so that the null hypothesis cannot be refuted.

To determine the significance of bonding technique on the permeability properties of hygiene nonwovens, a one-way ANOVA was used.

If the *p*-value is greater than 0.05 (i.e., *p* > 0.05 or *p* > 5%), the null hypothesis is accepted, which means that the differences between the classes are random. If the *p*-value is 0.05 or less, the result is considered significant and the null hypothesis is rejected, which means that there are differences between the classes and that these differences are statistically significant.

The ANOVA was performed with the statistical software SPSS Version: 29.0.0.0 (241) (IBM, London, UK) [40,41].

## 3. Results and Discussion

### 3.1. Results and Discussion of Breaking Stress and Elongation

The results of breaking stress and elongation are presented in Table 3.

The mechanically bonded samples exhibit the lowest breaking stress (from 0.519 N/mm^2^ to 1.448 N/mm^2^). In the case of mechanical bonding (spunlace bonding), there are no binders in the nonwoven and only the friction between the fibres in the nonwoven affects the lower tensile stresses, compared to the nonwoven with binders (in the case of thermal or chemical bonding). The binder in the chemical and thermal bonding processes causes a better bonding of the fibres themselves in the nonwoven structure and, thus, causes a higher strength compared to mechanical bonding (spunlace bonding), in which bonding is only achieved by mechanical stress and without the addition of binders.

Sample 7 exhibits the highest elongation at break (Table 3). The reason for the highest elongation at break for sample 7 lies in the bonding technique. Sample 7 is thermally bonded and has the highest surface openness (46.26%), the thickest fibre diameter (17.9 µm), and a very high thickness (0.34 mm), which affects the increase in elongation at break compared to the other samples (Table 3). Samples 3 and 4 exhibit the lowest elongation at break.

The reason for this is the smaller thickness of the samples (about 0.1 mm) and the average diameter of the fibres (about 13 μm). Both samples are also chemically bonded. Samples 3 and 4 are the thickest and have thinner fibres, which affects the decrease in elongation at break.

Samples 2 and 3, which are chemically bonded, have a very high breaking stress compared to the other specimens (37.92 N/mm^2^ and 50.81 N/mm^2^, respectively). Sample 5, which is mechanically bonded, has a very low breaking stress (0.519 N/mm^2^). The mass and thickness of Sample 5 is the highest (110.2 g/m^2^ and 0.98 mm), but the sample is mechanically bonded (spunlace). Sample 6, which is also mechanically bonded (spunlace), has a mass of 41.3 g/m^2^ and a breaking stress of 1.448 N/mm^2^.

Samples 4 and 7 also exhibit similar stresses (4.618 N/mm^2^ and 4.818 N/mm^2^). Sample 4 is chemically bonded and has a lower mass and thickness (0.17 mm and 26.8 g/m^2^) than Sample 7, which is thermally bonded. The similar breaking stress of Samples 4 and 7, with different mass and thickness, is due to the bonding technique. Sample 4 has a chemical binder on the surface, which influences the breaking stress. The thermally bonded Sample 7, with a mass of 39.2 g/m^2^ and a thickness of 0.34 mm, also consists of a binder and thermoplastic PP fibres.

The statistical analysis using one-way ANOVA shows the statistically significant influence of the bonding technique on the breaking stress and elongation.

### 3.2. Results and Discussion of Air Permeability

The results of air permeability are presented in Table 4.

The highest pore diameter and the highest surface openness allow a greater air flow through the nonwoven web and, consequently, allow a higher air permeability.

**Figure 2:**
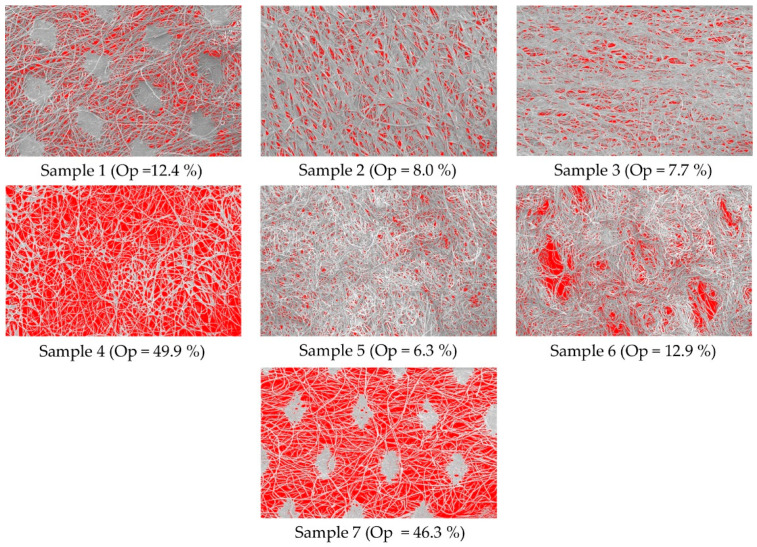
Process of image analysis and results of the surface openness.

**Figure 3:**
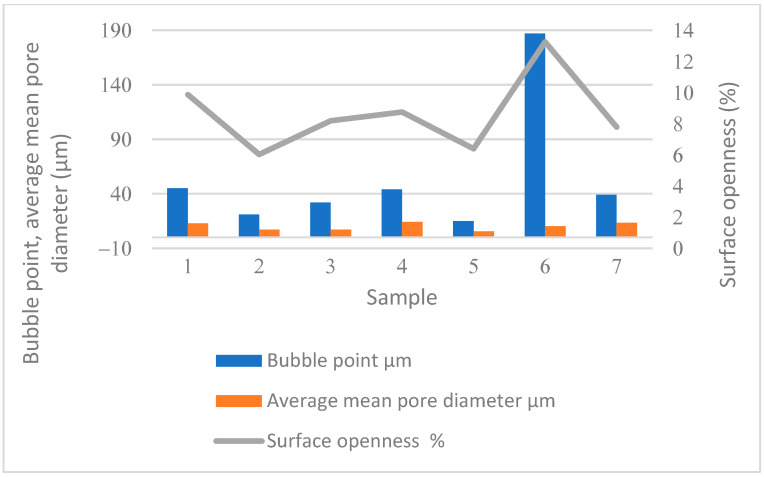
Results of porosity parameters.

Sample 4 has the highest air permeability, followed by Sample 1 (Table 4). Sample 4 has a very low thickness (0.17 mm), mass (26.8 g/m^2^), and the highest surface openness (49.87%, Figure 2). Sample 4, which is chemically bonded, also has the highest average mean pore diameter (14.41 μm, Figure 3).

Samples 1 and 7, with the second and third highest air permeability values (57 m^3^m^−2^min^−1^ and 47.45 m^3^m^−2^min^−1^), are both thermally bonded. In this case, the bonding fibres connect the other fibres in the nonwoven fabric only at the contact points (see Figure 1), which means that the other part of the structure remains open and air can flow through the nonwoven fabric, resulting in higher air permeability. Samples 1 and 7 also have a very high porosity.

Samples 2 and 3 have the lowest air permeability (approx. 15 m^3^m^−2^min^−1^). Samples 2 and 3 are chemically bonded with a mass of 50–70 g/m^2^. The surface openness of samples 2 and 3 is about 8%. The average pore diameter of Samples 2 and 3 is also the smallest (approx. 7 μm).

During chemical bonding, a binder is applied to the surface of the nonwoven web using various methods (calendering). The binder fills the empty spaces (pores) between the fibres and, thus, strengthens the structure. This makes the nonwoven fabric more rigid and less permeable to air. Figure 2 shows the distribution of the binder on Samples 2 and 3, which were chemically bonded.

The research results show that the bonding techniques (thermal, chemical, and mechanical) have a minor influence on the air permeability. A comparison of samples 1, 4, and 6, which were bonded thermally, chemically, and mechanically, with similar masses shows that Sample 4 has the highest air permeability. The reason for this is the higher surface openness and the thinner fibres of Sample 4 (chemically bonded).

**Figure 4:**
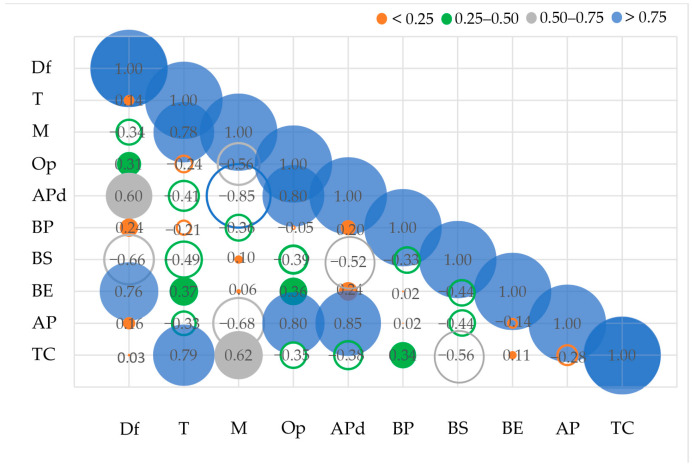
Correlation analysis between the presented parameters.

The statistical analysis using one-way ANOVA shows the statistically non-significant influence of the bonding technique on the air permeability, but, on the other hand, the bonding technique shows a statistically significant influence of the bonding technique on the porosity, which correlates strongly with the average mean pore diameter and the surface openness (Figure 4).

### 3.3. Results and Discussion of Thermal Conductivity

The results of thermal conductivity are presented in Table 5.

The contact points between the fibres also have adhesive points, which are mainly caused by friction between the fibres in the nonwoven. The mechanically bonded samples consist only of fibres that are joined together by mechanical force. Thermally and chemically bonded samples have a lower thermal conductivity than mechanically bonded samples. The bonding points between the fibres of the thermally bonded nonwoven samples and the binder applied to the surface of the chemically bonded nonwoven samples have an effect on the lower heat transfer through the nonwoven and, thus, on the lower thermal conductivity. Mechanically bonded samples have a smaller average mean pore diameter, which affects the smaller proportion of air trapped between the fibres, so that the thermal conductivity is higher for mechanically bonded nonwovens.

Table 5 shows that Sample 5 has the highest thermal conductivity (0.0804 Wm^−1^K^−1^), followed by Sample 6 (0.0749 Wm^−1^K^−1^). Samples 5 and 6 consist of 100% cotton fibres and are also mechanically bonded.

Samples 5 and 6 have a smaller average mean pore diameter, which affects the smaller proportion of air trapped between the fibres, so that the thermal conductivity is higher for mechanically bonded nonwovens.

The mass and thickness of Sample 5 are the highest (the mass is 110.2 g/m^2^ and the thickness is 0.98 mm), which means that the raw material composition and the bonding technique have a greater influence on the thermal conductivity than the mass and thickness, in this case.

Sample 3 has the lowest thermal conductivity (0.0573 Wm^−1^K^−1^). Sample 3 has the lowest thickness (0.09 mm). The thermal conductivity for all chemically bonded samples— 2, 3, and 4—is between 0.0573 Wm^−1^K^−1^ and 0.0643 Wm^−1^K^−1^. Sample 4, which consists of 100% viscose fibres, has a very similar thermal conductivity to Sample 2, which consists of 100% PP fibres (Table 5). Samples 2 and 4, with similar thermal conductivities, also have different masses (Table 5), but similar thicknesses (0.17 mm for Sample 4 and 0.20 for Sample 2).

Sample 3 is the most suitable as it has the lowest thermal conductivity and, therefore, the best insulating properties, compared to the other samples tested (Table 5).

If all the results on thermal conductivity are summarised, it can be said that the thermally and chemically bonded samples have a lower thermal conductivity, on average, than the mechanically bonded samples (spunlace).

The statistical analysis using one-way ANOVA shows the statistically significant influence of the bonding technique on the thermal conductivity.

### 3.4. Results and Discussion of Surface Openness

The results of the surface openness and the process of image analysis, as we obtained the values, is shown in Figure 2.

The results of the surface openness show that the mass of the sample and the diameter of the fibres have an important influence on the surface openness. The specific surface area of the thinner fibres of the nonwoven is higher, but when the mass is lower, the surface openness remains high.

Conversely, the nonwovens with thicker fibres and higher mass have a lower specific surface area and a higher surface openness. Sample 4 has the highest surface openness (49.87%), is chemically bonded, has the lowest mass (26.8 g/m^2^), and a very low thickness (0.17 mm) (Figure 2). Sample 4 has an average fibre diameter (13.7 µm) among the other samples. Sample 7, which also has a very high surface openness (46.26%), is thermally bonded. Sample 7 is thicker than Sample 4 and has a higher fibre diameter (17.9 µm) than Sample 4. The higher fibre diameter of Sample 7 also means a lower specific surface area of the fibres and, consequently, a higher surface openness. In addition to the bonding technique, the mass of the nonwoven and the fibre diameter also have an important influence on the surface openness.

Samples 1 and 6 have a similar surface openness (approx. 12%). Sample 1 is thermally bonded, while Sample 6 is mechanically bonded (spunlace). Both samples have a similar thickness (0.27 mm), while the diameter of the fibres of Samples 1 and 6 is between 15.7 µm and 17.9 µm.

Samples 2 and 3, which are chemically bonded, have a similar surface openness (from 7.74% to 7.96%).

The results show that Samples 2 and 3 have predominantly longitudinally oriented fibres, which influences the distribution of the binder between the fibres during chemical bonding and, thus, the openness of the surface.

Although Sample 3 has a smaller thickness (0.09 mm) and mass (49.8 g/m^2^), Samples 2 and 3 have a similar surface openness (7.96%—sample 2, 7.74%—sample 3). Sample 5, which is mechanically bonded, has the lowest surface openness. Sample 5 has a higher mass (110.2 g/m^2^) and thickness (0.98 mm) compared to the other samples tested. Sample 4 has the highest surface openness (49.87%), which is chemically bonded, while Sample 7, which also has a very high surface openness (46.26%), is thermally bonded. This means that the bonding technique has no influence on the surface openness.

The results of the surface openness show that the mass of the nonwoven and the fibre diameter have a large influence on the surface openness. The bonding technique has an important influence on the surface openness as it affects the number and shape of the contact points between the fibres. The surface openness is determined using the image analysis method, which is based on the surface structure of the nonwoven, while the porosity is based on the internal structure of the samples (number of pores, average pore diameter).

The results of the image analysis confirmed that the surface openness depends on the diameter of the fibres, the mass of the sample, and on the number and shape of contact points between the fibres. Samples 1 and 7 are both thermally bonded, but the number of contact points between the fibres is higher in Sample 1 (Figure 2). This is also the reason for the higher value of surface openness of Sample 7. The openness of the surface depends on the free parts of the fibres after bonding. Samples 2, 3, and 4 are chemically bonded, but Sample 4 has the highest surface openness, because the fibres of Sample 4 have more free parts after bonding (Figure 2).

### 3.5. Results and Discussion of Porosity

The results of the porosity are given in Table 6 and in Figure 3.

The results of the determined porosity parameters show that Sample 6 has a significantly larger pore diameter (bubble point); the largest pore diameter of Sample 5 is even 12 times smaller and is the smallest of all samples (Table 6 and Figure 3). Samples 5 and 6 are both mechanically bonded, but Sample 6 has a lower mass and thickness and, consequently, a higher bubble point and a larger average pore diameter. Sample 6 also has a smaller number of contact points between the fibres after bonding (Figure 2) and a greater surface openness than Sample 5.

Samples 2, 3, and 5 (5.7–7 μm); 1, 4, 6, and 8 (between 10 and 14 μm); and Sample 7 (19.6 μm) are similar in terms of the proportion of mean pore diameter—this should also be consistent with air permeability.

In Sample 1, approximately 36% of all pores are between 6 and 8 μm in diameter. Less than 1% or pores have a pore size of 30 μm or more. The maximum pore diameter is 45 μm. Sample 1 is thermally bonded. In this case, the binder fibres bind other fibres of the nonwoven at the contact points and the mass of the sample is very low. This results in an average pore diameter of 12.55 μm.

In Sample 2, about 28% of all pores are between 5 and 6 μm in diameter. Less than 1% of pores have a pore size of 14 μm and more. The maximum pore diameter is 21 μm. Sample 2 has similar porosity parameters to Sample 3. Samples 2 and 3 are both chemically bonded. In this case, the binder covers the surface of the nonwoven and influences a smaller average pore diameter (about 7 μm).

In Sample 3, about 48% of all pores are between 4 and 6 μm in diameter. Less than 1% of pores have a pore size of 18 μm or more. The maximum pore diameter is 32 μm.

In Sample 4, about 23% of all pores have a diameter between 9 and 10 μm. Less than 1% of pores have a pore size of 28 μm or more. The maximum pore diameter is 44 μm. Sample 4 has the second largest mean pore diameter (maximum, minimum, average, and mean pore diameter—Table 6) (between 12.4 and 15.8 μm) of all samples. Sample 4 has the lowest mass and diameter of the chemically bonded fibres, which influences the greatest surface openness and average mean pore diameter.

In Sample 5, about 35% of all pores are between 3 and 4 μm in diameter. Less than 1% of pores have a pore size of 11 μm or more. The maximum pore diameter is 15 μm, which is the smallest among all samples. In addition, Sample 5 has the smallest mean pore diameter (5.3 to 6 μm) of all samples. The reason for this is the higher mass and thickness of Sample 5. In Sample 6, about 80% of all pores are between 20 and 23 μm in diameter. Less than 1% of pores have a pore size of 60 μm or more. The maximum pore diameter is 187 μm, which is clearly the largest of all the samples. Samples 5 and 6 are both mechanically bonded, but Sample 6 has a lower mass and thickness and, consequently, a higher bubble point and a larger average mean pore diameter.

In Sample 7, approximately 25% of all pores are between 10 and 12 μm in diameter. Less than 1% of pores have a pore size of 40 μm or more. The maximum pore diameter is 59 μm. Sample 7 has the largest average pore diameter (from 15.7 to 23.4 μm) among all samples. Sample 7 is thermally bonded. In this case, the bonding fibres connect other fibres in the nonwoven at contact points and the mass of the sample is very low. This results in an average pore diameter of 13.56 μm.

The statistical analysis using one-way ANOVA shows the statistically significant influence of the bonding technique on the porosity.

### 3.6. Results and Discussion of Statistical Analysis

Summary statistics for the independent and dependent parameters are shown in Table 7, which lists the minimum, maximum, and average values of all measured parameters, to show the upper and lower limits of the analysed samples that we included in the study. As part of this study, we were also interested in the correlation between some physical properties of the samples (independent variables) and the observed parameters (dependent variables), so we also performed a correlation analysis between independent and dependent variables.

The correlations between the independent parameters, such as mass, thickness, fibre diameter, and surface openness, and the dependent parameters (average mean pore diameter, bubble point, breaking stress, breaking elongation, air permeability, and thermal conductivity) are shown in Figure 4.

The significance of mechanical, thermal, and chemical bonding on the mechanical and permeability properties of nonwovens for hygiene purposes was determined using one-way ANOVA (*p*-value < 0.05).

The results of the one-way ANOVA are shown in Table 8.

The correlation analysis shows a moderate negative correlation between fibre diameter, thickness, and breaking stress (Figure 4). There is a strong correlation between the diameter of the fibres and the elongation at break. There is also a strong correlation between the air permeability and the surface openness. The thermal conductivity correlates strongly with the thickness and moderately with the mass of the tested nonwovens.

The statistical analysis using one-way ANOVA shows the statistically significant influence of the bonding technique on the breaking stress and elongation, the thermal conductivity, and the porosity (*p*-value < 0.05) (Table 8). Statistical analysis, by means of one-way ANOVA, shows the statistically non-significant influence of the bonding technique on air permeability (*p*-value > 0.05) (Table 8).

## 4. Conclusions

In the present study, hygienic nonwovens were examined, which were mechanically, thermally, and chemically bonded.

From the results of the present study, it can be concluded that the bonding technique influences the breaking stress and elongation, the thermal conductivity, and the porosity, while the influence of the bonding technique on the air permeability is not significant.

The binder in chemical and thermal bonding causes a better bonding of the fibres in the nonwoven structure and, thus, results in a higher strength compared to mechanical bonding (spunlace), in which bonding is achieved only by mechanical stress and without the addition of binders. Other researchers have also confirmed that different treatments (plasma, binders) of fabrics increase the breaking stress [3].

When comparing the thermally, chemically, and mechanically bonded samples with similar masses, it can be seen that the samples with the largest surface openness, the smallest surface area, and the thicker fibres have the highest air permeability (chemically bonded samples).

If we summarise all the results for thermal conductivity, we can say that the thermally and chemically bonded samples have a lower thermal conductivity, on average, than the mechanically bonded samples (spunlace). The bonding points between the fibres of the thermally bonded nonwoven samples and the binder applied to the surface of the chemically bonded nonwoven samples influence the lower heat transfer through the nonwoven and, thus, results in a lower thermal conductivity.

On the other hand, mechanically bonded samples have a smaller average mean pore diameter, which influences the lower proportion of air trapped between the fibres, so that the thermal conductivity is higher in mechanically bonded nonwovens.

Other researchers also confirmed that the permeability properties of fabrics are mainly related to the pore size and porosity of the fibres [3]. The permeability properties also increase with a smaller surface area of the nonwoven fabric structure and the thickest fibres, as well as with the number of layers in the nonwoven fabric [4,5,6].

The research results of image analysis and porosity confirmed that the surface openness and porosity depend on the diameter of the fibres, the mass of the sample, and the number of contact points between the fibres and bonding technique. The surface openness and porosity depend on the free parts of the fibres after bonding. The samples with the highest surface openness, as well as a lower surface area and porosity, have more free fibre parts after bonding.

## Figures and Tables

**Table 1:** Analysed samples.

Sample	Web Bonding	Raw Material Composition	Nominal Mass (g/m^2^)
1.	Thermal process	70% polypropylene, 15% viscose, 15% cotton	30
2.	Chemical process	100% polypropylene	70
3.	Chemical process	100% polypropylene	50
4.	Chemical process	100% viscose	25
5.	Mechanical process	100% cotton	120
6.	Mechanical process	100% cotton	35
7.	Thermal process	100% polypropylene	40

**Table 2:** Diameter of fibres, thickness, and mass of analysed samples.

Sample	Diameter of Fibres (µm)	Thickness (mm)	Mass (g/m^2^)
1.	17.2	0.27	30.9
2.	13.3	0.20	74.2
3.	12.8	0.09	49.8
4.	13.7	0.17	26.8
5.	14.5	0.98	110.2
6.	15.7	0.27	41.3
7.	17.9	0.34	39.2

**Table 3:** Breaking stress and elongation of the analysed samples.

Sample	Breaking Elongation, ε (%)		Breaking Stress, σ (N/mm^2^)	
	Longitudinal	CV [%]	Longitudinal	CV [%]
1.	21.54	9.871	3.098	12.951
2.	15.85	8.137	37.922	9.631
3.	11.56	9.068	50.817	8.069
4.	9.86	7.350	4.618	9.046
5.	33.23	7.723	0.519	10.529
6.	27.44	8.950	1.448	13.250
7.	65.71	3.355	4.818	6.605

**Table 4:** The results of air permeability.

Sample	Air Permeability Q [m^3^m^−2^min^−1^]		
	Q [m^3^m^−2^min^−1^]	Sx [m^3^m^−2^min^−1^]	CV [%]
1.	56.75	2.34	4.12
2.	15.45	0.62	3.98
3.	15.10	1.63	10.76
4.	112.9	16.61	14.71
5.	12.11	1.18	9.74
6.	30.75	2.03	6.60
7.	47.45	1.63	3.45

Q—air permeability in m^3^m^−2^min^−1^; Sx—standard deviation; CV—coefficient of variation.

**Table 5:** The results of thermal conductivity.

Sample	Thermal Conductivity λ [Wm^−1^K^−1^]		
	λ [Wm^−1^K^−1^]	Sx [Wm^−1^K^−1^]	CV [%]
1.	0.0634	0.00032	0.507
2.	0.0643	0.0028	4.367
3.	0.0573	0.00023	0.410
4.	0.0635	0.0010	1.576
5.	0.0804	0.0010	1.244
6.	0.0749	0.0018	2.400
7.	0.0607	0.0021	3.502

λ—thermal conductivity in Wm^−1^K^−1^; Sx—standard deviation; CV—coefficient of variation.

**Table 6:** Results of porosity.

Sample	Maximum Mean Pore Diameter Ds_max_ (μm)	Minimal Mean Pore Diameter Ds_min_ (μm)	Average Mean Pore Diameter DS_avg_ (μm)	Maximum Pore Diameter —Bubble Point D_max_ (μm)
1.	15.15	10.78	12.55	45
2.	7.44	6.68	7.00	21
3.	7.69	6.47	7.22	32
4.	15.84	12.41	14.41	44
5.	6.00	5.32	5.61	15
6.	12.23	8.27	9.18	187
7.	14.93	11.95	13.56	39

**Table 7:** Summary statistics of parameters.

Variables	**	Observ.	Min.	Max.	Mean	Std.
Average mean pore diameter (µm)	APd	14	5.660	14.120	10.083	3.366
Bubble point (μm)	BP	14	15.000	187.000	54.714	57.090
Breaking stress (N/mm^2^)	BS	14	0.519	50.817	14.749	19.823
Breaking elongation (%)	BE	14	9.860	65.710	26.456	18.488
Air permeability (m^3^m^−2^min^−1^)	AP	14	12.110	112.900	41.500	35.870
Thermal conductivity (Wm^−1^K^−1^)	TC	14	0.057	0.080	0.066	0.008
Diameter of fibres (µm)	Df	14	12.800	17.900	15.014	1.898
Thickness (mm)	T	14	0.090	0.980	0.331	0.286
Mass (g/m^2^)	M	14	26.800	110.200	53.200	28.375
Surface openness (%)	Op	14	6.270	49.870	20.497	18.278

** APd—average mean pore diameter; BP—bubble point; BS—breaking stress; BE—breaking elongation; AP—air permeability; TC—thermal conductivity; Df—diameter of fibres; T—thickness; M—mass; Op—surface openness.

**Table 8:** Results of one-way ANOVA.

**ANOVA for Breaking Stress Results**						
**Source of Variation**	**SS**	**df**	**MS**	**F**	***p*-Value**	**F-Crit**
Between Groups (technology of bonding technique)	3309.4	2	1654.7	10.8	0.0012	3.68
Within groups	2278.9	15	151.9			
**ANOVA for Breaking Elongation Results**						
**Source of Variation**	**SS**	**df**	**MS**	**F**	***p*** **-Value**	**F-Crit**
Between Groups (technology of bonding technique)	2941.9	2	471.0	7.3	0.01	3.68
Within groups	3014.8	15	200.9			
**ANOVA for Air Permeability Results**						
**Source of Variation**	**SS**	**df**	**MS**	**F**	***p*** **-Value**	**F-Crit**
Between Groups (technology of bonding technique)	1544.1	2	1544.1	1.86	0.19	4.75
Within groups	9892.4	15	824.4			
**ANOVA for Thermal Conductivity Results**						
**Source of Variation**	**SS**	**df**	**MS**	**F**	***p*** **-Value**	**F-Crit**
Between Groups (technology of bonding technique)	0.00099	2	0.00049	64.92458	4.11 × 10^−8^	3.68
Within groups	0.00015	15	7.67 × 10^−6^			
**ANOVA for Porosity (Average Mean Pore Diameter) Results**						
**Source of Variation**	**SS**	**df**	**MS**	**F**	***p*** **-Value**	**F-Crit**
Between Groups (technology of bonding technique)	88.07	2.00	44.03	6.73	0.01	3.68
Within groups	98.20	15.00	6.55			

Sum-of-squares (SS) column with no repeated measures, degrees of freedom (df), mean squares (MS), F-ratio (F), *p*-value, F-critical (F-crit).

## Data Availability

The raw data supporting the conclusions of this article will be made available by the authors on request.
